# Association between short-term exposure to meteorological factors on hospital admissions for hemorrhagic stroke: an individual-level, case-crossover study in Ganzhou, China

**DOI:** 10.1265/ehpm.24-00263

**Published:** 2025-02-28

**Authors:** Kailun Pan, Fen Lin, Kai Huang, Songbing Zeng, Mingwei Guo, Jie Cao, Haifa Dong, Jianing Wei, Qiujiang Xi

**Affiliations:** 1 Department of Neurology, First Affiliated Hospital of Gannan Medical University, Ganzhou, 341000, China; 2 School of Public Health and Health Management, Gannan Medical University, Ganzhou, 341000, China; 3 Key Laboratory of Prevention and Treatment of Cardiovascular and Cerebrovascular Diseases, Ministry of Education, Gannan Medical University, Ganzhou, 341000, China; 4 School of the Frist Clinical Medicine, Gannan Medical University, Ganzhou, 341000, China

**Keywords:** Hemorrhagic stroke, Case-crossover design, Meteorological factors, Airborne particulate matter, Distributional lag nonlinear models

## Abstract

**Background:**

Hemorrhagic stroke (HS) is associated with significant disability and mortality. However, the relationship between meteorological factors and hemorrhagic stroke, as well as the potential moderating role of these factors, remains unclear.

**Methods:**

Daily data on HS, air pollution, and meteorological conditions were collected from January 2015 to December 2021 in Ganzhou to analyze the relationship between meteorological factors and HS admissions. This analysis employed a time-stratified case-crossover design in conjunction with a distributional lag nonlinear model. Additionally, a bivariate response surface modelling was utilized to further investigate the interaction between meteorological factors and particulate matter. The study also stratified the analyses by gender and age. To investigate the potential impact of extreme weather conditions on HS, this study defined the 97.5th percentile as representing extremely high weather conditions, while the 2.5th percentile was classified as extremely low.

**Results:**

In single-day lags, the risk of admissions for HS was significantly associated with extremely low temperature (lag 1–2 and lag 13–14), extremely low humidity (lag 1 and lag 9–12), and extremely high precipitation (lag 2–7). Females exhibited greater susceptibility to extremely low temperature than males within the single-day lag pattern in the subcomponent layer, with a maximum relative risk (RR) that was 7% higher. In the cumulative lag analysis, the risk of HS admissions was significantly associated with extremely high temperature (lag 0–8∼lag 0–14), extremely low humidity (lag 0–2∼lag 0–14), and extremely high precipitation (lag 0–4∼lag 0–14). Within the cumulative lag day structure of the subcomponent layer, both extremely low and extremely high temperature had a more pronounced effect on females and aged ≥65 years. The risk of HS admissions was positively associated with extremely high barometric pressure in the female subgroups (lag 0–1 and lag 0–2). The highest number of HS admissions occurred when high PM_2.5_ concentrations coexisted with low precipitation.

**Conclusions:**

Meteorological factors were significantly associated with the risk of hospital admissions for HS. Individuals who were female and aged ≥65 years were found to be more susceptible to these meteorological influences. Additionally, an interaction was observed between airborne particulate matter and meteorological factors. These findings contributed new evidence to the association between meteorological factors and HS.

**Supplementary information:**

The online version contains supplementary material available at https://doi.org/10.1265/ehpm.24-00263.

## 1. Introduction

Hemorrhagic stroke (HS) refers to intracranial hemorrhage resulting from the rupture of blood vessels in the brain, primarily characterized by varying degrees of altered consciousness and limb paralysis [1]. The type of HS encompasses intracerebral hemorrhage, subarachnoid hemorrhage, and non-traumatic intracranial hemorrhage [2]. Although it constituted only 15% of all stroke occurrences, HS was associated with a higher mortality and disability rate compared to ischemic stroke (IS) [3]. The economic burden associated with HS was significantly greater. In 2019, the per capita hospital costs for patients with IS and HS in China were 9,811.18 yuan and 19,843.37 yuan, respectively, reflecting average annual growth rates of 1.29% and 4.59% since 2004 [4]. HS is typically secondary to arterial rupture, which may be triggered by altered hypertension or other vascular anomalies [5]. The 30-day mortality rate for patients experiencing HS can reach up to 40%, and among those who survive, only approximately 20% achieve full functional autonomy six months following the HS episode [6]. Therefore, the identification and management of underlying risk factors remain key strategies for preventing HS, aiming to reduce its medical and economic burden.

Before examining the adverse effects of environmental factors on population health, previous studies have identified several primary risk factors for stroke, including high blood pressure, elevated cholesterol levels, obesity, smoking, and alcohol consumption [7, 8]. However, when environmental risk factors change, the body’s ability to effectively regulate its functions may be compromised. This disruption can disturb various regulatory mechanisms within the body, potentially leading to the onset and exacerbation of numerous serious diseases, as well as the aggravation of pre-existing conditions. Additionally, studies have indicated that the onset of stroke exhibited seasonal patterns, suggesting that variations in meteorological factors may be linked to the occurrence of HS [9, 10].

At the same time, extreme meteorological conditions increasingly elevated the risk of adverse health events [11]. Epidemiological evidence indicated that meteorological factors, including temperature, relative humidity, and barometric pressure, could influence the occurrence of HS [12–14]. Telman et al. conducted a study examining the relationship between HS and temperature in northern Israel, concluding that HS occurred more frequently in winter (n = 286, 29%; P < 0.01) [15]. This finding was consistent with results from a Danish study, which also determined that the likelihood of HS was elevated during the winter months when temperatures were lower [16]. A study conducted in Fujian, which analyzed data from two million patients across eight regions, found that a decrease in apparent temperature was associated with an increased risk of hospitalization for HS [17]. Additionally, another study demonstrated that the risk of HS rose with each 1% increase in relative humidity (OR: 1.000, 95% CI: 1.000–1.010) [18]. In addition to temperature and relative humidity, barometric pressure significantly influenced the morbidity and mortality associated with HS. A multicenter study conducted in Hiroshima, Japan, observed that elevated air pressure notably increased the risk of HS (RR = 1.31, 95% CI: 1.04–1.65) [19]. Xie et al. demonstrated that the risk of cerebral hemorrhage increased with higher concentrations of fine particulate matter (PM_2.5_) and inhalable particulate matter (PM_10_) [20]. Extensive research and evidence supported the independent effects of meteorological factors and airborne particulate matter on HS. However, in practice, the risk factors for HS typically do not exist in isolation; they often co-occur. The interaction between air pollutants and meteorological factors in relation to disease has been explored [21]. Several epidemiological studies suggested that the health effects of air pollution may be influenced by the meteorological environment [22]. However, the interplay between meteorological factors and atmospheric pollutants in the context of HS is complex, and the underlying biological mechanisms remain not fully understood [23]. Breton et al. indicated that the relative risk index of atmospheric pollution on population health increased significantly with every 5 °C rise in temperature in warm semi-arid regions [24]. Additionally, the effects of meteorological factors and atmospheric pollutants on health varied across different regions. In addition, most studies primarily focused on the interaction between temperature and pollutants [25, 26]; however, few have analyzed the interactions of precipitation, barometric pressure, relative humidity, and particulate matter on health. Due to the relatively low incidence of HS, few studies, including those conducted in the Ganzhou City, have specifically investigated the effects of airborne particulate matter and meteorological factors on this type of stroke. Furthermore, research examining the interaction effects related to HS was exceedingly rare.

This study presents a comprehensive study on the impact of short-term exposure to meteorological factors on HS, utilizing a time-stratified case-crossover design along with a distributional lag nonlinear model. Additionally, the interaction between meteorological factors and particulate matter concerning HS was examined through bivariate response surface modelling. The study also explored the potential moderating effects of two subgroups: gender and age, in relation to this association. Considering the continuous and lagged nature of exposure to meteorological factors and their effects, this study aimed to comprehensively assess the exposure-response relationship between both single and cumulative lag-day meteorological factor exposure and HS, along with its lagged effects. These findings not only aided in identifying high-risk susceptible populations but also provided a crucial epidemiological foundation for the development of effective preventive measures against HS.

## 2. Materials and methods

### 2.1. Data sources

This study was conducted at a single center, utilizing case data from one of the largest tertiary care hospitals in Ganzhou City, which accommodates over 1.57 million visits annually. Daily data on HS from January 1, 2015, to December 31, 2021, were collected based on the International Classification of Diseases, Tenth Revision (ICD-10) codes, including subarachnoid hemorrhage (I60), intracerebral hemorrhage (I61), and non-traumatic intracranial hemorrhage (I62). To minimize the impact of inaccuracies arising from coding, we verified the identified cases using the appropriate diagnoses. This dataset comprised information including disease codes, disease names, primary diagnoses, gender, age, and time of admission. Meteorological data were sourced from the National Meteorological Science Data Centre (https://data.cma.cn/), which included daily average temperature, daily average precipitation, daily average barometric pressure, and daily average relative humidity. Air pollution data were obtained from the Global Urban Air Quality Real-Time Dissemination Platform (http://air.cnemc.cn:18007), primarily encompassing the daily average values of PM_2.5_ and PM_10_.

### 2.2. Data analysis

In this study, we employed a time-stratified case-crossover design to develop a distributed lag nonlinear model (DLNM) based on a quasi-Poisson distribution, aimed at analyzing the nonlinear and lagged effects of meteorological factors on hospital admissions for HS. Furthermore, this design allowed for the adjustment of potential confounders (e.g., age, gender) through self-control, while also accounting for confounding variables such as seasonality and day-of-the-week effects through temporal stratification, thereby eliminating time trend bias [27]. In this study, the maximum number of lag days in the cross-basis was set to 14 to fully examine the lagged effect of meteorological factors. The model was expressed as follows:

$$\begin{array}{rl} \text{log}(E(Y_{t})) & =\alpha +\text{cb}(\text{Met},\text{df})+\text{ns}(\text{Pollution},\text{df})\\ & \;+\text{factor}(\text{holiday})+\text{factor}(\text{stratum}) \end{array}$$

*E*(*Yt*): expects number of HS cases on day t; *α*: the intercept equation; *cb*(): the cross-basis function; *ns*(): the natural cubic spline function of the nonlinear factor; *Met* is the meteorological factor; *Pollution*: the daily average PM_2.5_, PM_10_; *df*: the degrees of freedom; *factor*(*holiday*) is the dichotomous categorical for controlling for holiday effects variable; *stratum* is the time stratum variable. The degrees of freedom and the optimal model were determined using the Akaike Information Criterion (AIC) minimization principle, applied over a range of 3 to 5 degrees of freedom (Table S1 and S2). To avoid multicollinearity, factors with large correlation coefficients (|rs| > 0.7) were not included simultaneously in this study.

In this study, the potential impact of meteorological extremes on HS was explored by defining the 97.5th percentile as representing extremely high meteorological conditions, while the 2.5th percentile was considered to reflect extremely low meteorological conditions. For instance, the temperature at the 97.5th percentile was categorized as extremely high, whereas the temperature at the 2.5th percentile was categorized as extremely low. This approach was similarly applied to the extremes of other meteorological factors. Additionally, the study stratified analyses based on age (≥65 versus <65 years) and sex (male, female) to identify potentially sensitive populations. Finally, the effects of single lag days (lag 0∼lag 14) and cumulative lag days (lag 0–1∼lag 0–14) of meteorological exposure were examined for total cases as well as for specific subgroups. Z-tests were employed in this study to analyze whether statistically significant differences existed between the subgroups.

$$Z=\frac{\left(Q_{1}-Q_{2}\right)}{\sqrt{{\text{Se}}_{1}^{2}+{\text{Se}}_{2}^{2}}}$$

Q1 and Q2 represent the estimated effects of meteorological factors in the two subgroups of the population; *Se*_1_ and *Se*_2_: the corresponding standard errors, respectively.

In addition, this study employed a bivariate response surface model to analyze the interaction between meteorological factors and airborne particulate matter. Given that meteorological factors and airborne particulate matter differ in nature and units, we utilized a tensor product smoothing function (Te) to explore their interactions. By plotting the response surfaces among meteorological factors, airborne particulate matter, and health outcomes, we qualitatively assessed the significance of the interaction between the two variables. The basic model was:

$$\begin{array}{rl} \text{log}(E(Y_{t})) & =\beta +\text{Te}(k,x)+s(z)+\text{factor}(\text{holiday})\\ & \;+\text{factor}(\text{stratum}) \end{array}$$

*β* is the intercept; *k* is the value of meteorology on day t; *x* denotes the value of particulate matter concentration on day t; *S*(): the penalty spline function.

In this study, sensitivity analyses were conducted to assess the robustness of the findings. Firstly, the analysis examined the impact of meteorological factors on the results by adjusting their degrees of freedom, ranging from 3 to 5. Secondly, the study explored variations in the results by altering the maximum lag days of the meteorological factors, specifically at 6, 14, and 21 days. Additionally, the study primarily utilized the ‘splines’ and ‘dlnm’ packages in R version 4.2.3 to construct the model, applying a significance level of 0.05.

## 3. Results

### 3.1. Descriptive analysis of HS admissions profile and meteorological factors

Between January 1, 2015, and December 31, 2021, a total of 8,288 patients with HS were admitted to the hospital. The patient population comprised a higher number of males than females, with a male-to-female ratio of 1:0.63. The majority of patients were under the age of 65 years (67.51%) (Table 1).

**Table 1:** Demographic characteristics of study participants.

**Characteristics**	**Cases (%)**
Total	8288 (100)
Sex	
Male	5082 (61.32)
Female	3206 (38.68)
Age	
<65	5595 (67.51)
≥65	2693 (32.49)

**Fig. 1:**
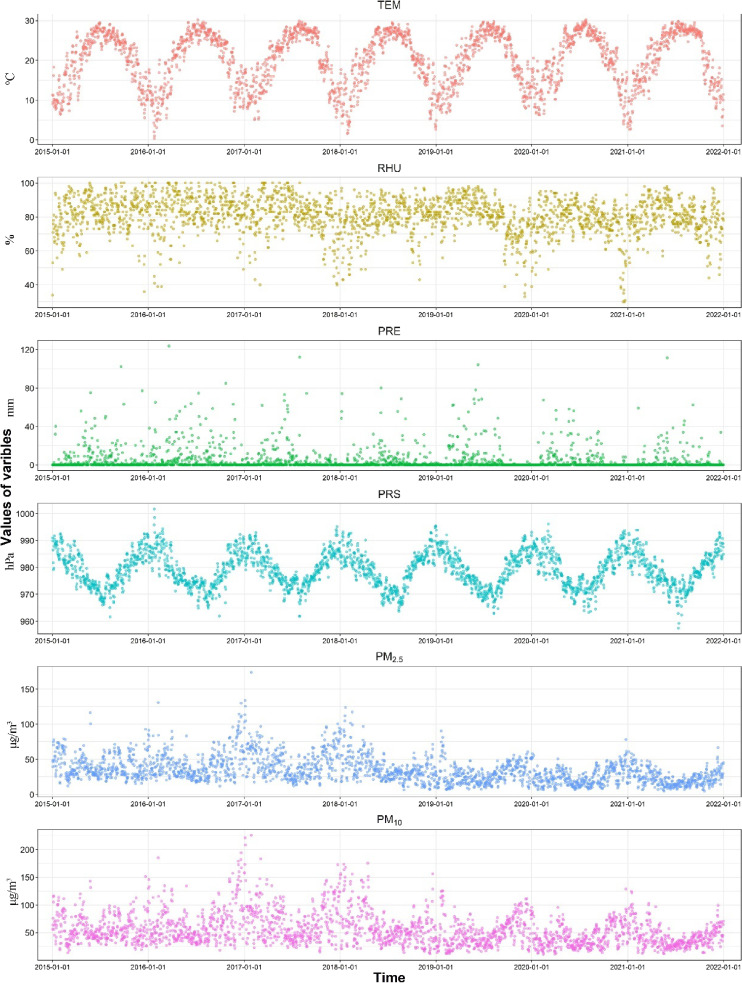
Time series plot of meteorological factors as well as airborne particulate matter in Ganzhou city. **Abbreviations:** TEM, temperature; RHU, relative humidity; PRE, precipitation; PRS, barometric pressure; PM_2.5_: fine particulate matter; PM_10_: inhalable particulate matter.

The median values for daily average temperature, daily average relative humidity, daily average precipitation, and daily average barometric pressure were 21.20 °C, 82.00%, 0.6 mm, and 978.90 hPa, respectively. The average concentration of PM_2.5_ was 34.82 µg/m^3^ (range: 4.56–174.25 µg/m^3^), while the average concentration of PM_10_ was 55.47 µg/m^3^ (range: 10.95–225.72 µg/m^3^) (Table 2). The time series analysis of meteorological factors and airborne particulate matter in Ganzhou City was illustrated in Fig. 1. The daily average temperature and daily average barometric pressure exhibited a distinct seasonal cycle, with fluctuations occurring in opposite directions.

**Table 2:** Basic information on airborne particulate matter and meteorological factors in Ganzhou, 2015–2021.

**Exposure**	**Min**	**25th**	**50th**	**75th**	**Max**	**Mean**	**Standard deviation**
Temperature (°C)	0.30	14.65	21.20	26.20	30.20	20.05	6.78
Relative humidity (%)	30.00	75.00	82.00	88.00	100.00	80.60	10.86
Precipitation (mm)	0.00	0.00	0.60	2.60	123.70	4.56	11.92
Barometric pressure (hPa)	957.50	974.00	978.90	984.10	1001.70	979.09	6.67
Fine particulate matter (µg/m^3^)	4.56	21.39	30.99	43.93	174.25	34.82	19.03
Inhalable particles (µg/m^3^)	10.95	34.80	49.03	69.35	225.72	55.47	29.16

### 3.2. Correlation analysis between meteorological factors and airborne particulate matter in Ganzhou City

**Fig. 2:**
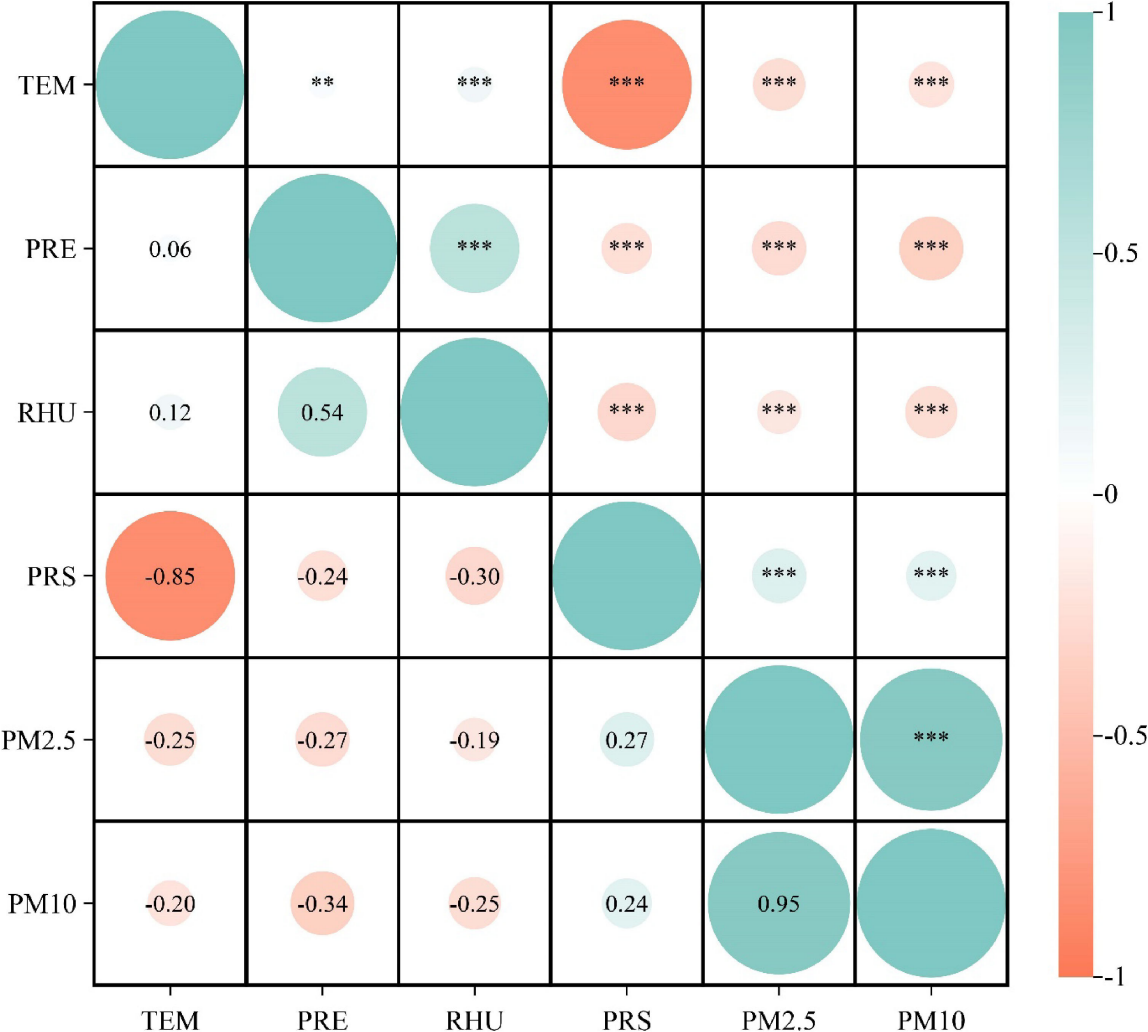
Spearman’s correlation coefficient plot of meteorological factors and airborne particulate matter in Ganzhou City, 2015–2021. * indicates 0.01 < P ≤ 0.05; ** indicates 0.001 < P ≤ 0.01; *** indicates P ≤ 0.001. **Abbreviations:** TEM, temperature; RHU, relative humidity; PRE, precipitation; PRS, barometric pressure; PM_2.5_: fine particulate matter; PM_10_: inhalable particulate matter.

Spearman analysis of meteorological factors and air pollutants in Ganzhou City showed that PM_2.5_ and PM_10_ were highly positively correlated (rs = 0.95, P < 0.001). PRS and TEM were highly negatively correlated (rs = −0.85, both P < 0.001). The correlations were all statistically significant (Fig. 2).

### 3.3. Single-day lag effects of extreme meteorological factors and airborne particulate matter in relation to HS

**Fig. 3:**
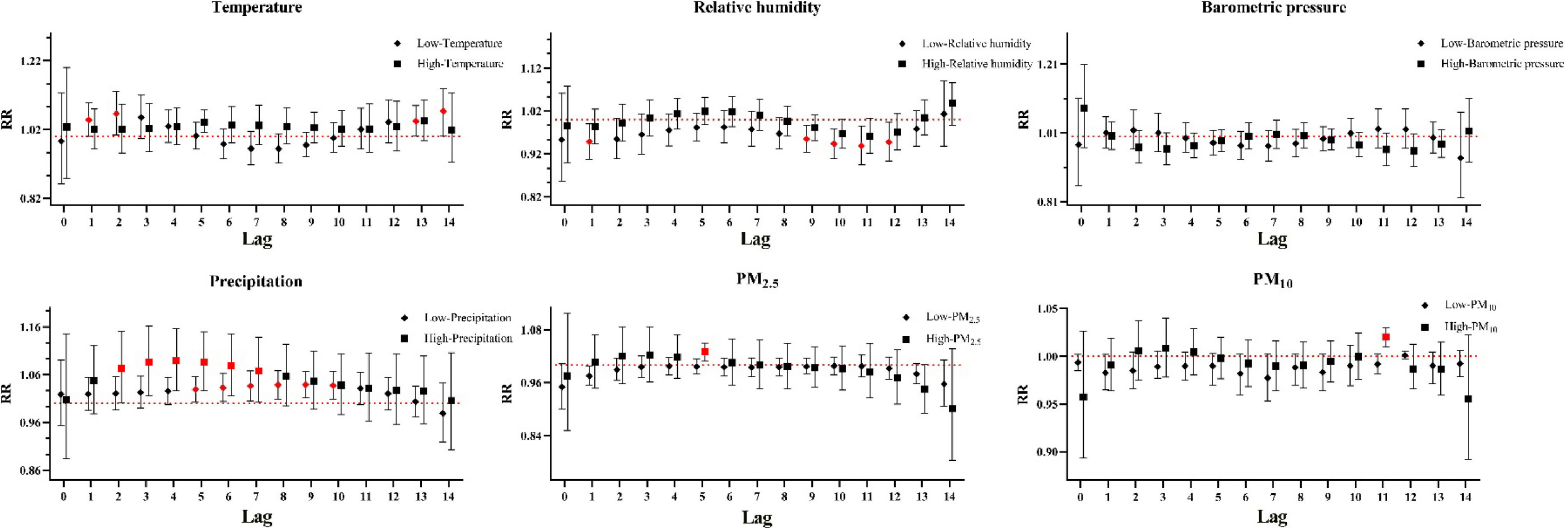
Single-day lag response curves of meteorological factors and airborne particulate matter on HS in total cases. Note: Red denotes a P < 0.05, Black denotes a P > 0.05.

The single-day lag response curves for total HS cases were shown in Fig. 3. The risk of admissions for HS was significantly associated with extremely low temperature (lag 1–2 and lag 13–14), extremely low humidity (lag 1 and lag 9–12), extremely high concentrations of PM_2.5_ (lag 5), extremely high concentrations of PM_10_ (lag 11), extremely high precipitation (lag 2–lag 7) and extremely low precipitation (lag 5–lag 10). Relative risk was found to be higher at lag 14 for extremely low temperature (RR = 1.07, 95% CI: 1.01–1.14), at lag 4 for extremely high precipitation (RR = 1.09, 95% CI: 1.03–1.16), at lag 5 for extremely high concentrations of PM_2.5_ (RR = 1.03, 95% CI: 1.01–1.05) and at lag 11 for extremely high concentrations of PM_10_ (RR = 1.02, 95% CI: 1.01–1.03). Conversely, extremely low humidity demonstrated the strongest protective effect against admissions for HS at lag 1 (RR = 0.95, 95% CI: 0.91–0.99). However, no significant association was observed between barometric pressure and HS within the single lag day structure.

**Fig. 4:**
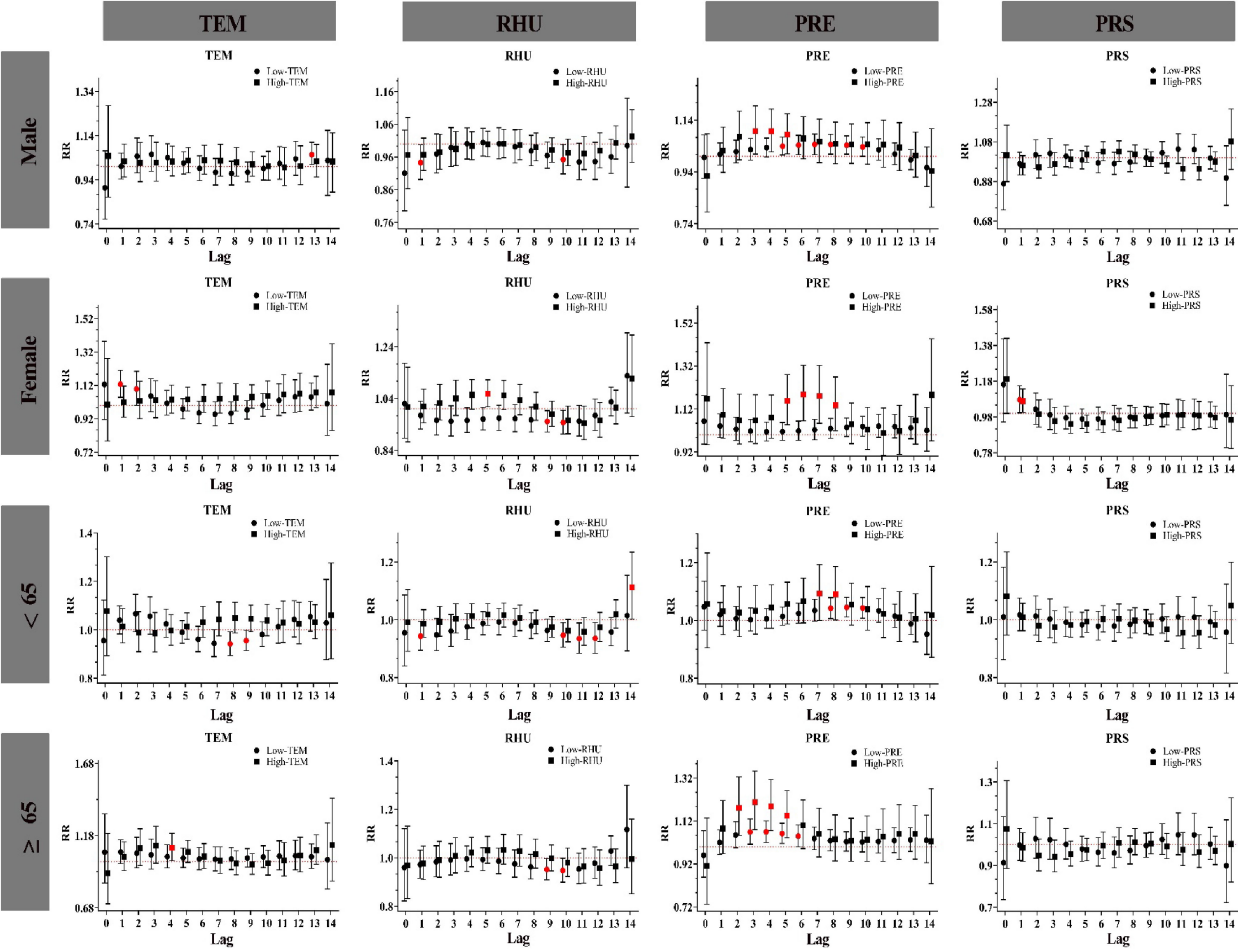
Single-day lag response curves of meteorological factors on subcomponent strata of HS. **Abbreviations:** TEM for temperature; RHU for relative humidity; PRE for precipitation; PRS for barometric pressure. Note: Red denotes a P < 0.05, Black denotes a P > 0.05.

Subgroup analyses were conducted based on sex and age to assess its moderating effect on the relationship between meteorological factors and HS. In the subgroup-stratified single-day lag pattern (Fig. 4), it was observed that women exhibited a greater susceptibility to extremely low temperature compared to men, with a maximum relative risk (RR) that was 7% higher. Extremely low relative humidity was significantly associated with an increased risk of admissions for HS in the male subgroup at lag 1 and lag 10, and in the female subgroup at lag 9 and lag 10. Meanwhile, extremely high precipitation was positively correlated with the risk of HS in the male subgroup at lag 3–5, and in the female subgroup at lag 5–8. The risk of HS was significantly associated with extreme barometric pressure (lag 1) exclusively in the female subgroup. In contrast, extremely low temperature demonstrated a protective effect against HS in the subgroups aged under 65 (lag 8–9). Extremely low relative humidity was significantly associated with an increased risk of HS across all four subgroups. Additionally, a significant risk effect was observed between extremely low precipitation and HS, with the exception of the female subgroup.

### 3.4 Cumulative lag effect of extreme meteorological factors and airborne particulate matter in relation to HS

**Fig. 5:**
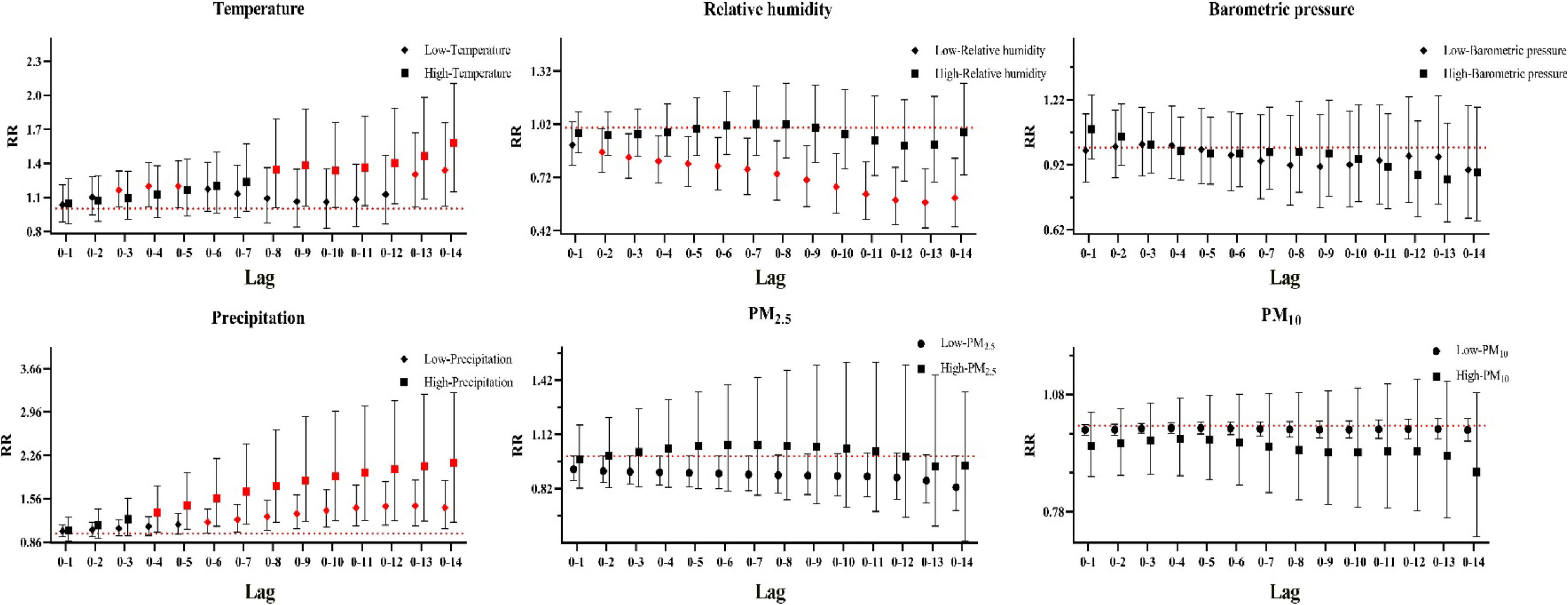
Cumulative lag response curves of meteorological factors and airborne particulate matter on HS in total cases. Note: Red denotes a P < 0.05, Black denotes a P > 0.05.

The cumulative effect response curves of extreme meteorological factors on the total number of HS cases were illustrated in Fig. 5. The risk of hospitalization for HS was significantly associated with extremely high temperature (lag 0–8∼lag 0–14), extremely low temperature (lag 0–3∼lag 0–5 and lag 0–13∼lag 0–14), extremely low relative humidity (lags 0–2∼lag 0–14), extremely high precipitation (lag 0–4∼lag 0–14), and extremely low precipitation (lag 0–6∼lag 0–14). Both extremely high and extremely low temperature exhibited the most significant hazard effect at lag 0–14 (RR = 1.58, 95% CI: 1.15–2.10; RR = 1.34, 95% CI: 1.02–1.76, respectively). Additionally, extremely low relative humidity was found to be protective against HS at both lag 0–2∼lag 0–14, with the protective effect being most pronounced at lag 0–2 (RR = 0.86, 95% CI: 0.75–0.99). The relative risk was highest for extremely low precipitation at lag 0–13 (RR = 1.45, 95% CI: 1.12–1.86) and for extremely high precipitation at lag 0–14 (RR = 2.14, 95% CI: 1.19–3.28). Conversely, the study did not find a significant effect of barometric pressure, PM_2.5_ and PM_10_ on HS.

**Fig. 6:**
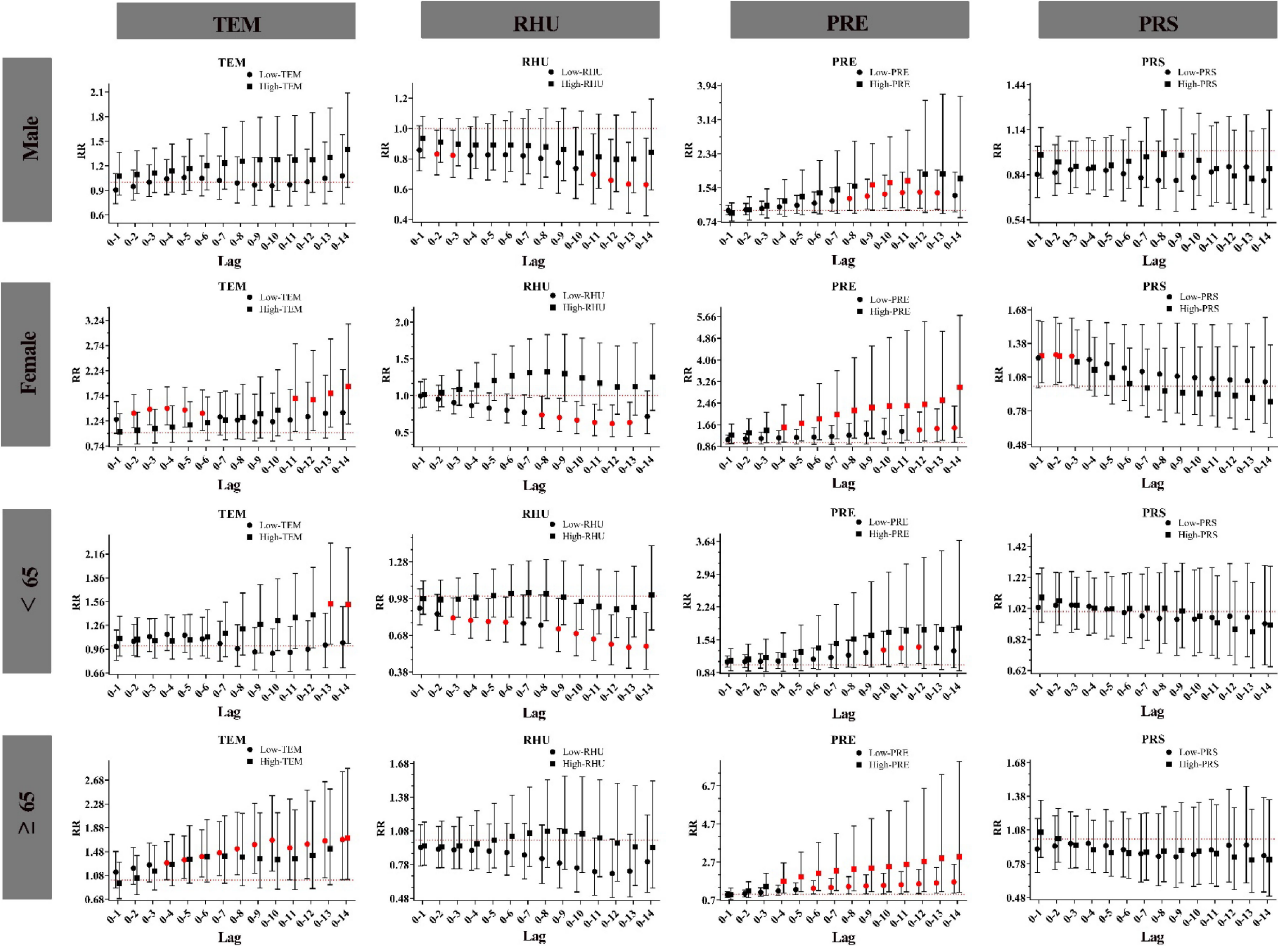
Cumulative lag response curves of meteorological factors on subcomponent strata of HS. **Abbreviations:** TEM for temperature; RHU for relative humidity; PRE for precipitation; PRS for barometric pressure. Note: Red denotes a P < 0.05, Black denotes a P > 0.05.

In the cumulative lag-day structure stratified by subgroup (Fig. 6), extremely low and extremely high temperature had a more pronounced effect on females and aged ≥65 years. The risk of admission for HS was positively associated with extremely high barometric pressure in the female subgroup (lag 0–1 and lag 0–2). A significant relationship was observed between extremely high precipitation and HS, with the exception of the subgroup aged under 65. However, this study did not find any effect of extremely high humidity on HS.

### 3.5 Interaction between meteorological factors and particulate matter on admissions for HS

**Fig. 7:**
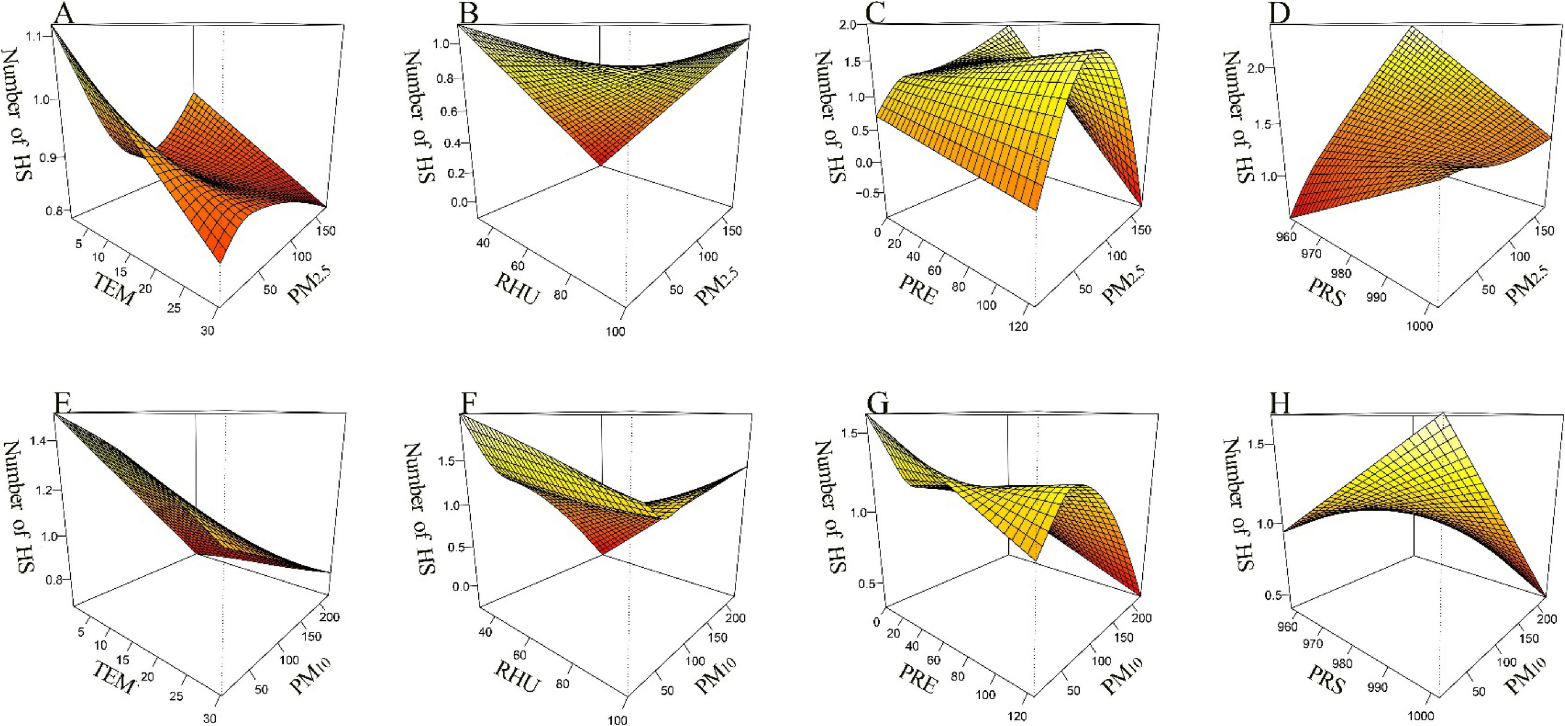
Interaction between meteorological factors and airborne particulate matter on admissions for HS. **Abbreviations:** TEM, temperature; RHU, relative humidity; PRE, precipitation; PRS, barometric pressure; PM_2.5_: fine particulate matter; PM_10_: inhalable particulate matter.

Bivariate response surface models were employed to illustrate the interaction analysis between meteorological factors and airborne particulate matter concerning HS admissions (Fig. 7). The number of HS admissions exhibited a gradual peak as temperature decreased, particularly under conditions of high PM_2.5_ and low PM_10_ concentrations. The number of admissions for HS peaked at low PM_2.5_-low relative humidity and low PM_10_-low relative humidity. The number of admissions for HS was highest when elevated concentrations of PM_2.5_ coincided with low precipitation levels, as well as when low concentrations of PM_10_ were present alongside low precipitation. Additionally, the number of HS admissions rosed with decreasing barometric pressure in the context of high concentrations of both PM_2.5_ and PM_10_.

### 3.6. Sensitivity analysis

In this study, sensitivity analysis was employed to assess the findings and evaluate the robustness of the model. By varying the degrees of freedom for the meteorological factors (ranging from 3 to 5) and the maximum lag days (6, 14, and 21), it was observed that the trend remained relatively stable. This indicated that the model exhibited a higher degree of robustness (Fig. S1 and S2).

## 4. Discussion

Previously, several studies have analyzed the effects of air pollutants and meteorological factors on HS; however, the conclusions drawn were inconsistent and fail to consider the interaction between these two factors in relation to the disease. Therefore, this study aimed to assess the effect of meteorological factors on HS, examine the moderating effect of subgroups on these relationships, and further explore the interaction between meteorological factors and airborne particulate matter, using the city of Ganzhou as the study area. In this time-series analysis, the study identified an adverse effect of meteorological factors on 8,288 hospitalizations due to HS from 2015 to 2021. Furthermore, the analysis revealed that the association between HS and meteorological factors was influenced by gender and age. Overall, individuals who were female and aged ≥65 years exhibited greater susceptibility to meteorological factors, and an interaction was observed between airborne particulate matter and meteorological conditions.

A Japanese study indicated that outdoor temperature was significantly and negatively associated with the risk of HS [28]. Conversely, McDonald et al. found no correlation between changes in meteorological factors and the incidence of HS in a survey conducted in the United States [29]. A multicenter study conducted in Jiangsu Province indicated that heat exposure elevated mortality rates from stroke and HS by 1.54% (95% CI: 1.44%–1.65%) and 1.36% (95% CI: 1.26%–1.48%) respectively, and that females and the elderly were more susceptible to the effects of heat exposure [30]. A multicentre time-series study conducted by He et al. in Shandong found that 12.55% of HS mortality was attributed to non-optimal temperature, suggesting that exposure to both low and high temperatures increased the risk of death from HS, which was consistent with the findings of the present study [31]. Cold exposure has been reported to activate both the sympathetic nervous system and the renin-angiotensin system, leading to an increase in blood pressure and a predisposition to HS [12, 32]. However, the mechanism by which high temperature influences the occurrence of HS remains unconfirmed. This study concluded that extremely low temperature had a more detrimental effect on HS at a single-day lag, whereas extremely high temperature were more harmful at a cumulative lag. This finding aligned with the research conducted by Luo et al., which also indicated that extremely low temperature were most harmful to HS on the day of lag [33]. The discrepancies in these results are challenging to interpret and may arise from the possibility that shorter lag periods may not adequately capture the potentially harmful effects of high temperature [34]. The various characteristics of the study cities, including climate resilience, housing types, air conditioning penetration, and population vulnerability, were noteworthy [35]. Furthermore, the differences in study designs, health outcomes (mortality versus morbidity), and modelling strategies may have also influenced the variability of our findings compared to previous reports.

A survey conducted in Italy by Biondi et al. [36] revealed that the likelihood of stroke increased in environments characterized by low humidity and high PM_10_ concentrations. Conversely, the onset risk of stroke and cerebral hemorrhage was found to be negatively correlated with relative humidity, as reported in studies conducted in the USA [37] and Switzerland [38]. Additionally, research by Myung et al. in Korea also indicated a negative correlation between the occurrence of HS and relative humidity [39]. These findings were consistent with the results of the present study. The increased likelihood of exposure to dust or toxic substances in humid air may amplify the impact of relative humidity on disease risk when considered in isolation regarding its health effects [40]. Further studies are needed to explore the biological mechanisms by which relative humidity influenced the risk of hospitalization for HS, as well as to investigate the differences across various population subgroups.

There were relatively few reports examining the correlation between precipitation and HS. A study conducted by Matsumoto et al. on the cumulative effects of meteorological factors on stroke incidence found that the incidence of stroke increased with higher amounts of rainfall (OR = 1.59, 95% CI: 1.15–2.20) [41]. This finding aligned with the results of the present study. Extreme precipitation can impair the body’s ability to perspire in humid environments, thereby hindering the discharge of sodium and chloride ions, which may contribute to hypertension [42]. In turn, hypertension served as an independent risk factor for the development of HS, potentially increasing the risk of its occurrence by nearly tenfold [43].

To develop more targeted intervention strategies, it was essential to identify potentially sensitive populations. This study found a stronger association between meteorological factors and specific groups, including women and patients aged ≥65 years. Notably, meteorological factors were more closely linked to the number of HS visits among women compared to men. This finding aligned with the research conducted by Ma et al., which suggested that females were more susceptible to heat waves [44]. Stroke was the fifth leading cause of death for men and the third leading cause of death for women. Additionally, social factors, such as living habits and economic conditions, differed between women and men. These differences may make the weather have an impact on their health [45–47].

Individuals aged ≥65 years who experienced HS were particularly vulnerable to the adverse effects of temperature and precipitation. Research conducted by Chen and Yin has demonstrated that both cold spells and heat waves contributed to increased mortality rates among the elderly population [48, 49]. Furthermore, a study by Tang et al. indicated that individuals aged ≥65 years were more susceptible to the impacts of extreme precipitation compared to those aged <65 years [50]. This phenomenon may be attributed to the fact that older individuals exhibited lower body resistance and a diminished capacity to adapt to change in the external environment. Furthermore, they were more susceptible to underlying diseases, and the progressive aging of cerebral blood vessels increased their risk of HS [51].

Response curves indicated an interaction between meteorological factors and airborne particulate matter concerning HS; however, studies exploring these interactions were limited and uncertain. Qin et al. demonstrated that at elevated temperature, each 10 µg/m^3^ increase in PM_10_ corresponded to a 2.40% rise in non-accidental mortality among the total population (95% CI: 0.64% to 4.20%) [25]. As reported by Cheng et al., the interaction between high PM_2.5_ levels and cold waves has been shown to have attenuated effects on human health [52]. This finding was consistent with the results of the present study. Additionally, Qiu et al. found that cold and dry conditions significantly increased the risk of HS associated with PM_2.5_ and PM_10_ exposure [53]. Research has shown that blood pressure tended to rise in low humidity conditions. Additionally, pollutants that entered the body through various biological reactions may exacerbate this increase in blood pressure, potentially leading to HS [54–56]. When extremely low temperature and high concentrations of PM_2.5_ or PM_10_ occured simultaneously, individuals tended to reduce their time spent outdoors and adopt enhanced self-protection measures to effectively mitigate the harm caused by environmental risk factors [57, 58]. Conversely, when these two factors occured in isolation, vulnerable populations failed to take timely precautions to adapt to the stress induced by rapid changes in the external environment, potentially resulting in cardiovascular and cerebrovascular diseases [59].

This research presents specific strengths. Firstly, a time-stratified case-crossover design was employed in conjunction with the DLNM method to investigate the link between meteorological influences and admissions for HS. The case-crossover approach is well-regarded as a robust technique for evaluating the immediate effects of meteorological variables on health, while the DLNM effectively captures the non-linear and delayed impacts of these factors on HS hospitalizations. In addition, this research conducted a qualitative analysis of the relationship between meteorological variables and airborne particulate matter through the use of bivariate response surface modelling, offering a novel viewpoint on strategies for disease prevention and management. Furthermore, this study identified a delayed impact of meteorological conditions on HS and investigated the at-risk population for this condition via subgroup analysis. These results indicated that both society and families ought to focus more on and safeguard these susceptible groups.

This research presents several limitations as well. To begin with, it employed an ecological study design, which restricted the ability to draw causal inferences. Furthermore, the results may be affected by additional risk factors, including other individual stroke risk factors and behaviour patterns. This study focused exclusively on three types of HS and did not include an analysis of subdural and epidural haematomas, highlighting the need for further research to investigate the risk of stroke across various HS subtypes. Furthermore, the potential modifying effects of variables such as hypertension, diabetes, smoking, alcohol consumption, and family history of stroke were not evaluated in this study due to a significant amount of missing data for these variables. Finally, the findings were derived from data collected at a single hospital, which was the largest tertiary hospital in Ganzhou City. However, caution was warranted when generalizing these results to other regions.

## 5. Conclusions

This study demonstrated that short-term exposure to meteorological factors was significantly associated with the risk of HS admissions. Additionally, gender and age moderated the adverse effects of meteorological factors on the risk of HS admissions. Individuals who were female and aged ≥65 years were more susceptible to meteorological factors, resulting in a higher risk of HS. Additionally, there existed an interaction between air pollutants and meteorological factors. This study offered new evidence regarding the association between meteorological factors and HS, highlighting the need for further research to assist governments in effectively mitigating the impact of meteorological factors on the residents.

## Abbreviations

HS: Hemorrhagic stroke
TEM: temperature
RHU: relative humidity
PRE: precipitation
PRS: barometric pressure
PM_2.5_: fine particulate matter
PM_10_: inhalable particulate matter

## Supplementary information

**Additional file 1:** **Table S1** Selection table for degrees of freedom for meteorological factors.

**Additional file 2:** **Table S2** Selection table for degrees of freedom for particulate matter.

**Additional file 3:** **Fig. S1.** Plot of results of changing varieties degrees of freedom.

**Additional file 4:** **Fig. S2.** Plot of results for changing the maximum lag days for varieties.

## Declarations

### Availability of data and material

Available upon reasonable request.
