# Supplementary material for: Association between short-term exposure to meteorological factors on hospital admissions for hemorrhagic stroke: an individual-level, case-crossover study in Ganzhou, China
Source: Environ Health Prev Med. 2025 Feb 28;30:12. doi: 10.1265/ehpm.24-00263 (PMC11875774; doi:10.1265/ehpm.24-00263)
Supplement: Supplementary file 1 — Additional file 1: Table S1 Selection table for degrees of freedom for meteorological factors. [file ehpm-30-012-s001.docx]

| **Table S1** Selection table for degrees of freedom for meteorological factors. | | | | | |
| --- | --- | --- | --- | --- | --- |
| Dependent variable | Meteorological factor | The df of atmosphere | The df of PM2.5 | The df of PM10 | AIC |
| Cases | TEM | 3 | 3 | 3 | 11039.17 |
|  | RHU | 4 | 3 | 3 | 11052.13 |
|  | PRE | 4 | 3 | 3 | 11067.13 |
|  | PRS | 3 | 3 | 3 | 11085.41 |
| Male | TEM | 3 | 3 | 3 | 9532.812 |
|  | RHU | 4 | 3 | 3 | 9541.286 |
|  | PRE | 4 | 3 | 3 | 9539.903 |
|  | PRS | 3 | 3 | 3 | 9545.988 |
| Female | TEM | 3 | 3 | 4 | 8029.995 |
|  | RHU | 3 | 3 | 4 | 8029.297 |
|  | PRE | 4 | 3 | 4 | 8045.232 |
|  | PRS | 3 | 3 | 4 | 8042.802 |
| ＜65 | TEM | 3 | 3 | 3 | 9836.065 |
|  | RHU | 4 | 3 | 3 | 9826.003 |
|  | PRE | 4 | 3 | 3 | 9848.604 |
|  | PRS | 3 | 3 | 3 | 9856.576 |
| ≥65 | TEM | 3 | 3 | 3 | 7439.569 |
|  | RHU | 3 | 3 | 3 | 7471.535 |
|  | PRE | 4 | 3 | 3 | 7456.793 |
|  | PRS | 3 | 3 | 3 | 7482.343 |

**Abbreviations:** TEM, temperature; RHU, relative humidity; PRE, precipitation; PRS, barometric pressure; PM2.5: fine particulate matter; PM10: inhalable particulate matter.
