# Supplementary material for: Association between short-term exposure to meteorological factors on hospital admissions for hemorrhagic stroke: an individual-level, case-crossover study in Ganzhou, China
Source: Environ Health Prev Med. 2025 Feb 28;30:12. doi: 10.1265/ehpm.24-00263 (PMC11875774; doi:10.1265/ehpm.24-00263)
Supplement: Supplementary file 2 — Additional file 2: Table S2 Selection table for degrees of freedom for particulate matter. [file ehpm-30-012-s002.docx]

| **Table S2** Selection table for degrees of freedom for particulate matter. | | | | |
| --- | --- | --- | --- | --- |
| Airborne particulate matter | The df of particulate matter | The df of temperature | The df of relative humidity | AIC |
| PM2.5 | 3 | 3 | 3 | 11078.97 |
| PM10 | 3 | 3 | 3 | 11093.70 |

**Abbreviations:** PM2.5: fine particulate matter; PM10: inhalable particulate matter.
