# Supplementary figures and images for: Association between short-term exposure to meteorological factors on hospital admissions for hemorrhagic stroke: an individual-level, case-crossover study in Ganzhou, China
Source: Environ Health Prev Med. 2025 Feb 28;30:12. doi: 10.1265/ehpm.24-00263 (PMC11875774; doi:10.1265/ehpm.24-00263)

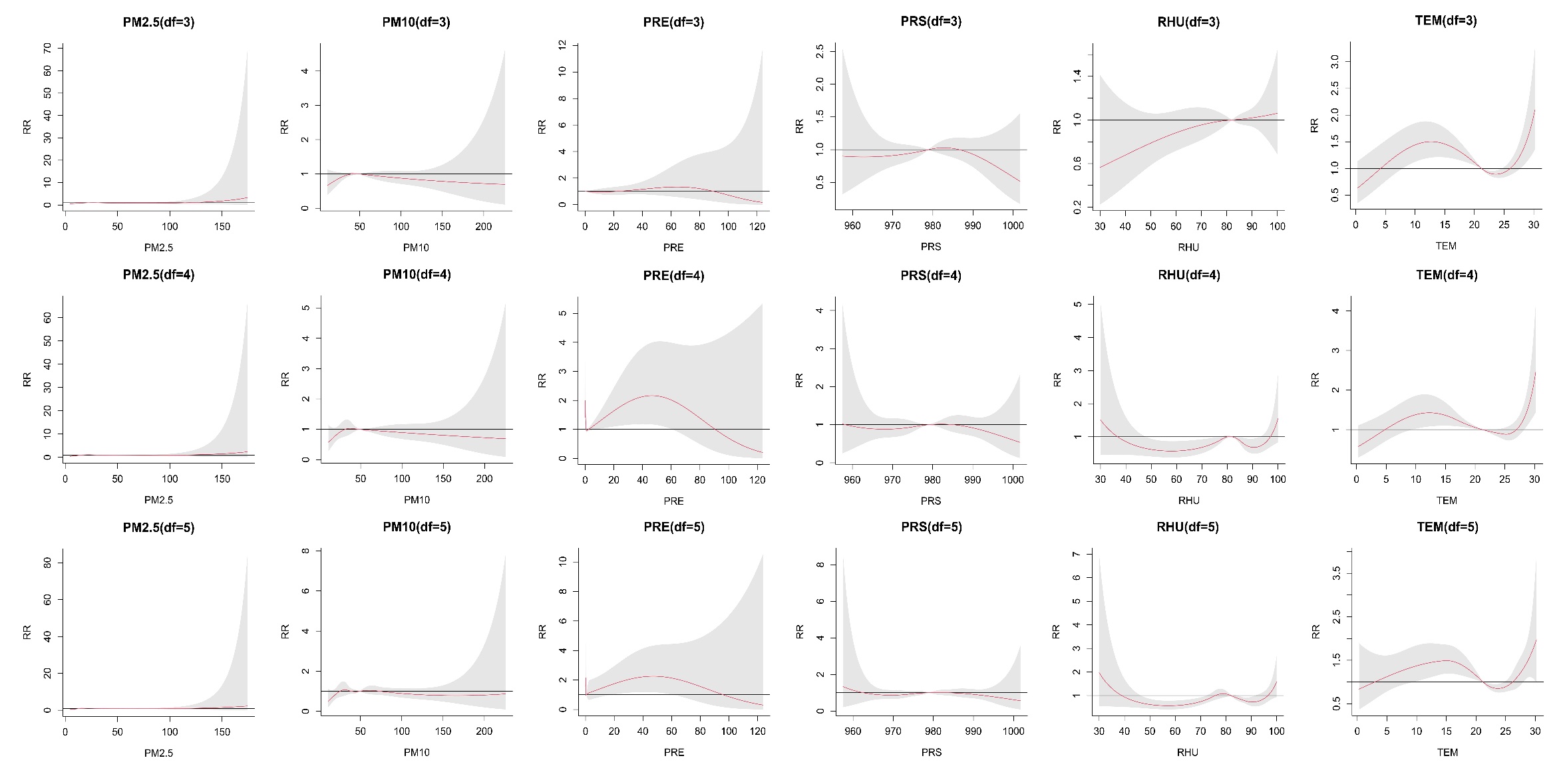
**Fig. S1.** Plot of results of changing varieties degrees of freedom.

Supplement: Supplementary file 3 — Additional file 3: Fig. S1. Plot of results of changing varieties degrees of freedom. [file ehpm-30-012-s003.docx]

**Fig. S2.** Plot of results for changing the maximum lag days for varieties.
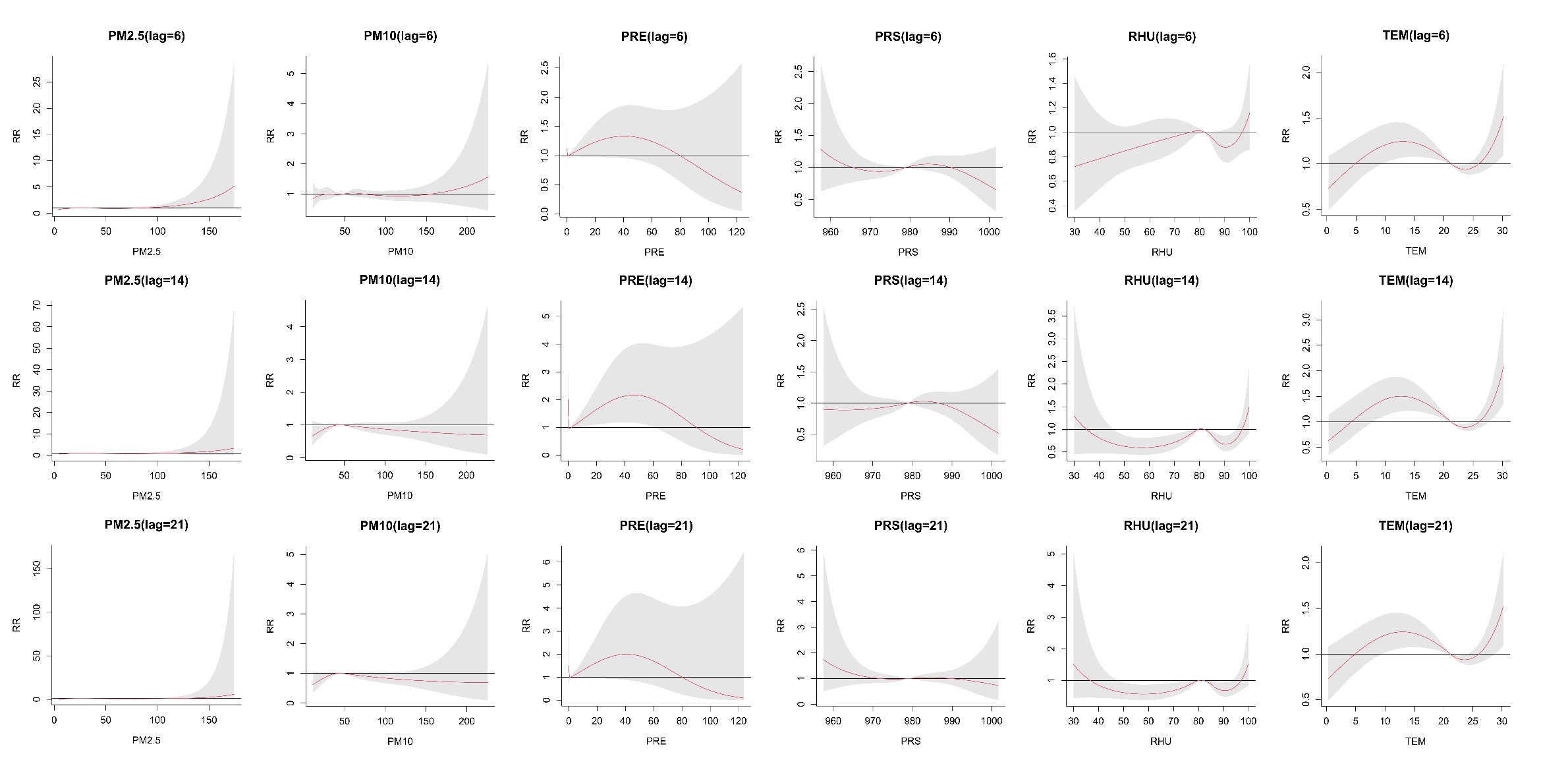

Supplement: Supplementary file 4 — Additional file 4: Fig. S2. Plot of results for changing the maximum lag days for varieties. [file ehpm-30-012-s004.docx]
